# Current modalities of sickle cell disease management

**DOI:** 10.1097/BS9.0000000000000056

**Published:** 2020-08-27

**Authors:** Adekunle Sanyaolu, Ejoke Agiri, Carl Bertram, Latasha Brookes, Jesy Choudhury, Dorina Datt, Amira Ibrahim, Anna Maciejko, Anna Mansfield, Jasmine Nkrumah, Martina Williams

**Affiliations:** a Federal Ministry of Health, Abuja, Nigeria; b Saint James School of Medicine, Anguilla, BWI

**Keywords:** Hemoglobin S (HbS), Sickle cell anemia, Sickle cell disease (SCD), United States of America

## Abstract

Sickle cell disease (SCD) affects nearly 100,000 people in the United States of America and the sickle gene is present in approximately 8% of black Americans. Among Africans, the prevalence of sickle cell trait (heterozygosity) is as high as 30%. While SCD occurs among varying racial and ethnic groups, it is more commonly prevalent in individuals of African or African-American descent. This inherited blood disorder causes varying symptoms and complications among affected children and adults and early diagnosis and treatment are essential to help reduce mortality rates. Because there is no cure for SCD, management is vital to survival. Hence, there are different approaches in use to aid those living with SCD; thus, this paper provides insight into the current methods that are implemented in the management and maintenance of this disease.

## 1 INTRODUCTION

**Figure 1:**
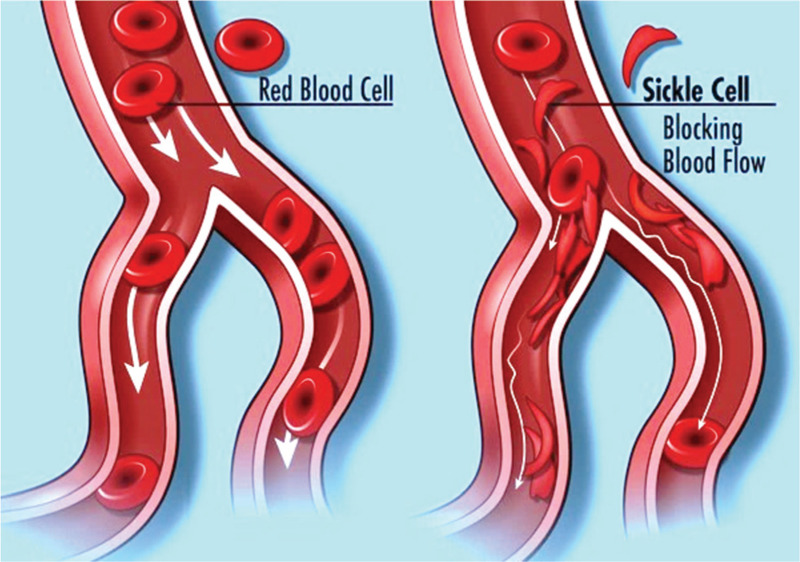
Normal red blood and sickle cells. *Source*: National Library of Medicine (US). Genetics Home Reference [Internet]. Bethesda (MD): The Library. Available at: https://ghr.nlm.nih.gov/. Accessed February 12, 2019.

Sickle cell disease (SCD) is a serious, inherited condition in which the body's erythrocytes (which contain hemoglobin and are responsible for the transport of oxygen throughout the body) become crescent or sickle-shaped, due to a mutation in the *HBB* gene.^1^ As a result, an abnormal beta-globin molecule is produced, called hemoglobin S (HbS).^1^ HbS replaces both beta-globin subunits in hemoglobin^1^ and arises as a cause of an anomaly in nucleotide substitution, resulting in the placement of valine instead of glutamate.^2^ The expression of hemoglobin S elicits a conformational change in the red blood cells, from round to sickle-shaped, making them harder and stickier,^3^ as depicted in Figure 1. Because of these changes, they tend to accumulate and adhere to the walls of small blood vessels, obstructing blood flow, and causing various health issues in children and adults such as sickle cell anemia.^2–4^

Other complications associated with SCD are more prevalent in children than adults.^5^ These include dactylitis and Hand-foot syndrome, growth retardation, delayed sexual maturation, being underweight, increased susceptibility to infections, aplastic crisis, vaso-occlusive events, splenic sequestration and infarction,^6^ strokes and injury to the brain such as a silent cerebral infarct, and acute chest syndrome.^2,5,7–9^ In teenagers and adults, other complications may include but are not limited to, damage to the lungs, heart, and kidneys as well as to the eyes.^5^ Priapism may also develop, along with gallstones, leg ulcers, deep vein thrombosis, pulmonary embolism,^8^ neurological impediments, chronic haemolysis, pulmonary artery hypertension and complications arising from frequent blood transfusions.^7^

Due to the varying complexities of these complications, whereby some can become life-threatening,^7^ there are many treatment options available to help relieve patients of their extreme discomfort.^10^ Not only do therapeutic modalities lead to better well-being of a patient, but they also provide probable ways to help in the containment of complication recurrence as well as reduce mortality rates.^11^ Furthermore, by considering improvements and alternative solutions to current treatment methods, adjustments can be made to ensure that patients receive the most effective care available to them. This has prompted the need for this study.

## 2 GLOBAL EPIDEMIOLOGY OF SCD

Millions of people throughout the world are affected by SCD.^12^ SCD occurs most commonly in regions where malaria is widespread, where the interaction between them is destructively synergistic.^13^ The highest prevalence occurs among people of African, African-American, Mediterranean (Italian, Sicilian, Greek), Middle Eastern, East Indian, Caribbean, and Central or South American descent.^12^ Migration of substantial populations from these high prevalence countries to Europe have dramatically increased in recent decades.^12^

Sickle cell disease originated in West Africa, where it has the highest prevalence.^2,12^ It is also present to a lesser extent in India and the Mediterranean region.^2,12^ Until recently, it was accepted that due to DNA polymorphism of the beta S gene, it is suggested that sickle cell disease arose from 5 separate mutations: 4 in Africa and 1 in India and the Middle East.^2^ However, a recent study based on modeling of balancing selection of the heterozygote advantage showed that the mutational change in the red corpuscle is caused by a single base substitution in β-globin which arose approximately 7300 years ago in West Africa.^14,15^ The selective force involved in the genetic spread which occurred precisely 4000 or more years ago in India and 3000 years ago in Africa during the agriculture expansion was most likely the malaria parasite *Plasmodium falciparum*.^14,15^ There is a selective advantage in the heterozygous form and this accounts for the high prevalence and persistence of the hemoglobin S gene.^2^ Due to the concordance that exists between the prevalence of malaria and hemoglobin S, it is believed that sickling protects a person from malaria by either accelerating sickling so that parasitized cells are removed, or making it more difficult for the parasite to metabolize or to enter the sickle cell.^2^ While children with the sickle cell trait seem to have a milder form of falciparum malaria, those with homozygous hemoglobin S have a severe form that is associated with a very high mortality rate.^2^

### 2.1 Prevalence of SCD worldwide

Africa has 3-quarters of the highest number of cases of sickle cell disease.^16,17^ In several sections of Africa, the prevalence of sickle cell trait (heterozygote) is as high as 30%.^16^ Six (6) to 9 million infants are born each year with sickle cell disease in Africa.^16^ According to the World Health Organization, around 2% of newborns in Nigeria were affected by sickle cell anemia (Table 1).^16^ In Nigeria alone, a total of 150,000 affected children are born every year.^16^ Across equatorial Africa, the carrier frequency ranges between 10% and 40%.^16^ Studies have shown a significant reduction in infant mortality rate, especially those aged 2–16 months, because of the sickle-cell trait in areas that were known to be major areas of malarial cases.^12^

**Table 1:**
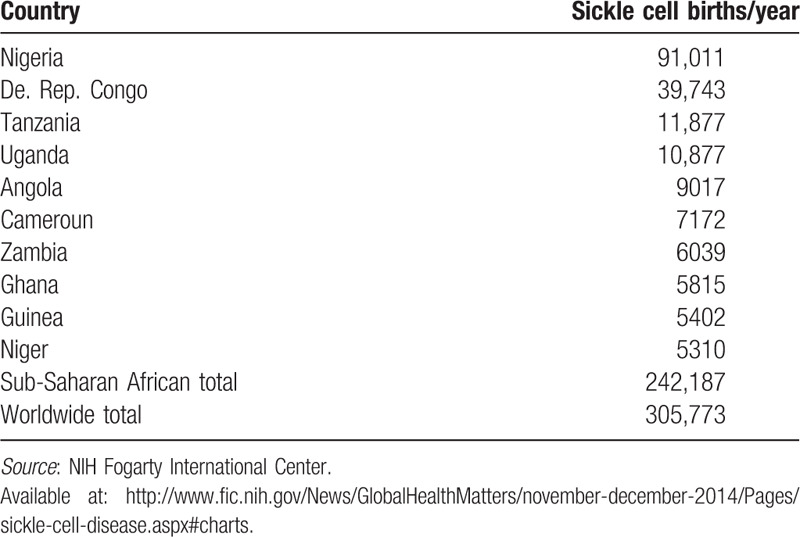
Number of sickle cell births per year according to country.

**Figure 2:**
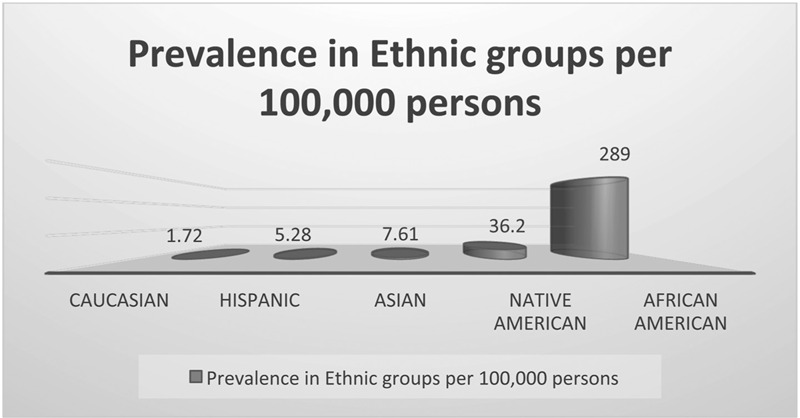
Prevalence of sickle cell disease in the United States among various ethnic groups. *Source*: The Caribbean Current. Available at: https://thecaribbeancurrent.com/sickle-cell-anaemia-is-it-a-black-disease/.

According to the National Institute of Health, the prevalence of the disease in the United States is approximately 1 in 5000, mostly affecting Americans of Sub-Saharan African descent.^2^ Within the United States, there are 2 million carriers and 72,000 people affected by SCD.^2^ SCD is present mostly in blacks, occurring in about 1 out of every 500 Black or African-American births (Fig. 2).^2^ The sickle gene is present in approximately 8% of black Americans.^2^ The expected prevalence of sickle cell anemia in the United States is 1 in 625 persons at birth.^2^ Approximately 4000 to 5000 pregnancies are at risk for sickle cell disease each year in the U.S.^2^ The actual prevalence is less because of early mortality.^2^ Although, the true number of individuals with SCD in the US remains unknown, populations will continue to be quantified by estimation due to the absence of a reliable surveillance system.^18^ It is estimated that the number of individuals with SCD may be between 104,000 and 138,900, with a mean estimate of 119,100 based mainly on the use of birth cohort-disease prevalence as applied to contemporary U.S. at-risk and total populations. After correction was made for early mortality associated with sickle cell anemia, population estimates were reduced to between 72,000 and 98,000. Among the African-American birth cohort, the estimate of the overall prevalence of SCD was (1:365), and that of sickle cell anemia was (1:601).^18^

The median age of death is now 53 in men and 58 in women for SCD.^16^ This dramatic decrease in mortality is due to newborn screening and improved comprehensive care.^16^ Nevertheless, there is still an increase in hospitalizations in the fifth decade of life with 48% of surviving patients resulting in severe organ damage.^16^ In developing countries, however, access to comprehensive medical care is poor.^16^ For instance, there are more than 20,000 infants that are born with SCD every year.^16^ With scarce access to health care, death is commonly seen in early childhood.^16^ In Saudi Arabia, with a population of over 23 million, the status of SCD carriers ranges from 2% to 27%.^16^

## 3 MANAGEMENT OF SICKLE CELL DISEASE

### 3.1 Current diagnostic procedures

#### 3.1.1 Newborn screening tests

Mandatory screening at birth for HbS uses prenatal tests that can be done by obtaining samples from the amniotic fluid or the placenta.^19^ Samples are screened for the sickle hemoglobin gene.^19^ Testing before birth can be performed as early as 10 weeks after pregnancy.^19^ SCD is commonly diagnosed with gel-based or capillary electrophoresis, high-performance liquid chromatography (HPLC), and isoelectric focusing. The tests are limited by their inability to reliably distinguish HbSS from HbS/β^0^-thalassemia. Other sophisticated tests with high sensitivity include tandem mass spectrometry, deoxyribonuclease acid (DNA) diagnostics (including Taqman polymerase chain reaction (PCR) and sequence analysis of specifically amplified *HBB* genes), and next-generation sequencing analysis. Several point-of-care tests are also currently under development; the most advanced is an antibody-based lateral-flow assay called Sickle SCAN which is now available commercially.^14^

#### 3.1.2 Electrophoresis

A blood test at any time during a person's lifespan can be taken to check if the sickle cell trait is present.^20^ Early detection is necessary and most of the time the blood test is part of the newborn screening.^18^ The blood test checks for hemoglobin S, the form of hemoglobin that is defective in sickle cell anemia.^20^

In babies, the blood test is usually taken from the finger or heel.^21^ In adults, the blood test is taken from the vein of the arm.^22^ If the screening test is negative, then the patient does not have a sickle cell.^22,23^ If the screening test is positive, another test is taken to determine if either 1 or 2 genes are present.^23^ If 1 gene is present, then the patient has the sickle cell trait and a small amount of hemoglobin S.^24^ If the patient has 2 genes they have sickle cell anemia and have a large amount of abnormal hemoglobin.^24^ Electrophoresis confirms diagnosis with tests positive for homozygous HbS.^25^

#### 3.1.3 Blood tests

Blood samples are taken and examined with a microscope (peripheral blood smear) to look for sickle cells, a marker for the disease.^26^ If a patient has the disease, then a blood test for anemia is performed.^20^ They are also analyzed for complete blood count (CBC) with reticulocyte and differential count, serum electrolytes, and hemoglobin solubility testing.^27^ A comprehensive metabolic panel is also done to assess renal function by looking at the levels of creatinine, and BUN as well as hepatobiliary function by observing the amount of bilirubin and ALT present.^27^ Pulmonary function tests are also performed.^27^ Usual baseline blood abnormalities in patients with SCD are hemoglobin level of 5–9 g/dL, decreased hematocrit of 17%–29%, elevated total leukocyte count of 12,000–20,000 cells/mm^3^ (12–20 × 10^9^/L), with a predominance of neutrophils, increased platelet count, low erythrocyte sedimentation rate, elevated reticulocyte count which may vary depending on the extent of baseline hemolysis, demonstrated target cells, elongated cells, and characteristic sickle erythrocytes in peripheral blood smear, Presence of red blood cells containing nuclear remnants (Howell-Jolly bodies) indicating that the patient is asplenic, positive hemoglobin solubility testing result that does not distinguish between sickle cell disease and sickle cell trait.^2^

#### 3.1.4 Imaging

Imaging studies that help with the diagnosis of sickle cell disease include: radiography, MRIs, computed tomography (CT) scans and transcranial Doppler (TCD) ultrasonography.^28^

Radiographic results may reveal soft tissue swelling in those with dactylitis, coupled with the formation of new periosteum which can be observed 7–10 days after onset.^29^ In a patient with osteonecrosis, secondary to sickle cell anemia, x-ray images may reveal joint degeneration, initial patchy sclerosis, and bone flattening.^29^ Furthermore, expansion of the medullary cavities, thinning of the cortices, trabecular resorption, as well as focal lucency, may also appear on radiographic imaging 2–3 weeks following symptom outbreak.^29^

Magnetic Resonance Imaging (MRI) is the preferred approach involving the early detection of bone marrow changes.^29^ Decreased signal intensity on T1- and T2-weighted MRI scans may indicate the presence of chronic bone infarcts and/or fibrosis.^29^ Furthermore, hyperplasia of the bone marrow may also be observed, whereby widespread, intermediate signal intensity appears on the images of T1- weighted scans.^29^ This method of imaging has a 98% specificity and 85%–97% sensitivity involving the identification of bone marrow infarcts.^29^

Computed tomography scans help to determine the presence of osteomyelitis.^29^ Periosteal reaction, destruction of bone, sinus tracts and dead bone can be observed using this method of imaging.^29^ However, this is not the preferred approach involving the assessment of acute osteomyelitis.^29^

Transcranial Doppler ultrasonography is used to determine the risk of stroke in patients with SCD.^30^ TCD is a safe, non-invasive method that determines intracerebral blood flow by the use of a pulsed Doppler transducer.^31^ This transducer emits resonant waves, taking in the reflections of the erythrocytes’ surface that are located within the intracranial vessels.^31^ The gathered results are examined through a computer, yielding numerical and visual data, which is used to ascertain the blood flow within the cerebral vessels.^31^ By localizing a cranial window, the intracranial arteries are assessed based on resonation depth, the direction of blood flow in reference to the probe and the waveform of the TCD.^31^ Measures of the peak systolic and diastolic velocities are taken, along with mean flow velocity as well as the Gosling pulsatility index (PI), where calculations are performed, aiding in the diagnosis process.^31^ Although this method is non-invasive and has beneficial potential, it also has its limitations,^30,31^ both of which are outlined in Table 2:

**Table 2:**
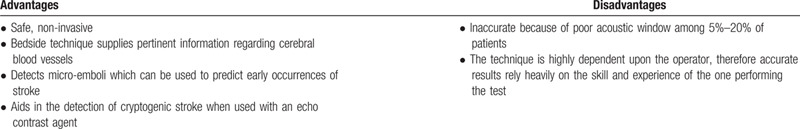
Advantages and disadvantages of transcranial Doppler ultrasound^29^

In one study of 38 asymptomatic children with SCD, investigators found that hypertension and abnormal blood pressure patterns were prevalent in children with SCD.^32^ They suggested using 24-hour ambulatory BP monitoring (ABPM) to identify these conditions in young patients.^32^ In the study, 17 patients (43.6%) had ambulatory hypertension, whereas 4 (10.3%) had hypertension based on their clinic blood pressure.^32^ Twenty-three patients (59%) had impaired systolic blood pressure dipping, 7 (18%) had impaired diastolic blood pressure dipping, and 5 (13%) had reversed dipping.^32^ If left undetected and untreated, hypertension and abnormal blood pressure levels may give rise to various health complications.^2,7,32^

### 3.2 Current treatment and management

**Figure 3:**
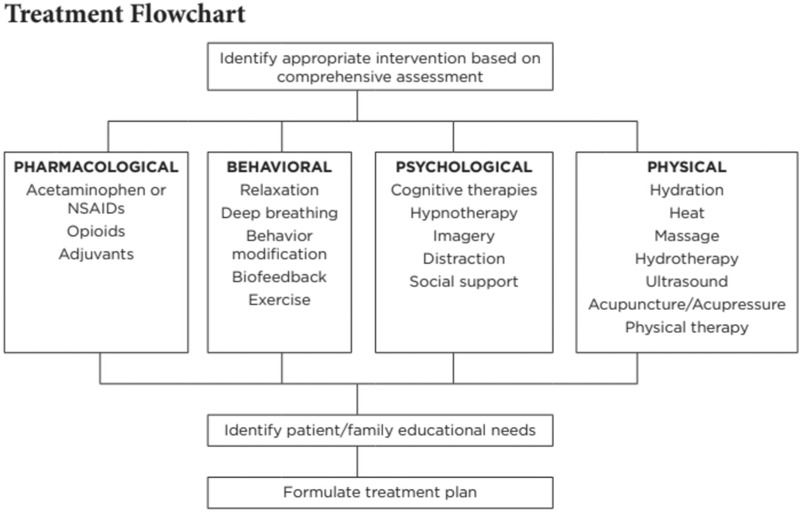
Factors to consider in the formulation of a treatment plan. *Source*: Sickle Cell Disease Critical Elements of Care [Internet]. Available at: http://cshcn.org/sites/default/files/webfm/file/CriticalElementsofCare-SickleCell.pdf. Accessed February 12, 2019.

Treatment procedures may be variable depending upon the circumstances, symptoms, and complications present in the patient.^2,7^Figure 3 outlines the factors to consider in the formulation of a treatment plan.

#### 3.2.1 Prophylaxis/supportive therapy

Genetic screening for sickle cell trait and disease can be tested in 2 manners: The first being a blood test using HPLC method, which can check for hemoglobin S.^33^ Secondly, sickle cell disease can be diagnosed by sampling amniotic fluid for the sickle cell gene from the pregnant mother.^34,35^ Although sickle cell trait (SCT) is not considered to be a health problem, individuals who test positive should be informed about the implications for their health and family planning.^35,36^ When a newborn with SCT is identified through screening, at least one of the parents will have SCT, and pregnant women during her prenatal care, from racial groups with a high prevalence of the sickle cell gene, are frequently tested for the gene.^36^ SCT counseling has 2 components; education and decision-making.^36^ The focus is on education, to enable individuals to make informed decisions, in their interest, about future family planning.^36^

#### 3.2.2 Management of SCD related pain

An acute painful crisis is the hallmark of SCD. It is the main cause of hospitalization for the disease; the precipitating triggers are both known and unknown. The painful crisis is responsible for the majority of damage to SCD patients.^37^ As the patients’ transition into adulthood, the pain episodes, and utilization of the health-care system become more frequent. Pain is a major day-to-day symptom for adults with the disease.^37^ The 2014 Expert Panel Report provides a treatment algorithm to address acute pain episodes in SCD patients and their recommendations include the rapid initiation of analgesics (within 30 minutes of triage or 60 minutes of registration). Also recommended were nonsteroidal anti-inflammatory drugs for mild-to-moderate pain and parenteral opioids for patients with severe pain.^37,38^ The pain should be reassessed and opioids can be administered every 15–30 minutes till the pain is reduced based on the patient report; the dose may be escalated by 25%. Other recommendations include hydration, use of nonpharmacologic approaches such as heat, and use of oral antihistamines when required for itching secondary to opioid administration.^37,38^

#### 3.2.3 Prophylaxis antibiotics for SCD

Individuals with SCD have a higher susceptibility to acquiring pneumococcal infections due to their risk of developing functional asplenia,^39^ which can occur as early as 6 months of age.^7,37^ Once the functional ability of the spleen is absent, the organ can no longer serve its immunological functions of clearing bacteria from the blood and synthesizing antibodies.^40^ This loss of function can, therefore, increase the chance of developing infections.

In a study administered by the *PR*ophylaxis with *O*ral *P*enicillin in children with *S*ickle cell anemia (PROPS), infants and children with sickle cell anemia between 3 months to 3 years of age received prophylactic penicillin.^41^ Results showed an 84% decrease in pneumococcal infection rates.^41^ A second study known as PROPS II was administered to evaluate the consequences of discontinuing penicillin prophylaxis at 5 years of age.^42^ Based on the PROPS and PROPS II results, there were no differences in regards to the rate of infection in the penicillin group compared with the placebo arm (relative risk = 0.5).^40^ However, problems with penicillin prophylaxis emerge and involve compliance, drug cost, patient tolerance, and resistant strains of micro-organisms.^40–42^

Subsequent studies have shown improvement in survival upon the administration of penicillin prophylaxis to individuals with sickle cell anemia.^43^ Administration of penicillin prophylaxis significantly decreases the risk of septicemia and death and is recommended for all children starting at 2 months of age.^44^

#### 3.2.4 Hydroxyurea and other medications

In adults, hydroxyurea is used in the treatment of recurrent and painful vaso-occlusive episodes by downregulating the ET-1 gene expression in endothelial cells.^45,46^ It also reduces the ability of HbS to adhere to the walls of the blood vessels, thereby enabling the return of sufficient blood flow throughout the body.^46^ Children with sickle cell disease treated with hydroxyurea have been shown to have levels of circulating ET-1 that was 2 times lower than those of untreated SCD children.^47^ It improves clinical outcomes by increasing fetal hemoglobin (HbF), which in turn, reduces risks of sickling events.^47^ Hydroxyurea, a ribonuclease reductase inhibitor, is the most disease-modifying agent of all current therapies for managing SCD. Many pediatric and adult trials have reported a reduction in pain, acute chest syndrome, and overall mortality. Patients must be carefully monitored for toxicity, especially early in therapy and the drug should not be used for the treatment of acute pain. Patient adherence is an issue with the drug since the lag of 3–6 months between initiation and the patient experiencing a clinical response often lead to nonadherence by patients.^37,48^ Also, conflicting evidence of the effects of Hydroxyurea on male fertility exists.^49^

#### 3.2.5 Bone marrow transplant

There has been success in children with bone marrow transplantation.^50^ This is done by using chemotherapy drugs to destroy all the bone marrow and have it replaced by new bone marrow by a donor.^51^ The only drawback to this procedure and cure is that the patient will have to be on immunosuppressant medications for months to a couple of years depending on the rejection of the allograft.^51^ These immunosuppressant medications can have mild to severe complications when taken routinely for long periods.^51^ The pre-procedural chemotherapy and post-procedural immunosuppressant medications were thought to be too toxic for adults.^51^ Recently, large organizations such as NIH, National Institute of Diabetes and Digestive and Kidney Diseases (NIDDK) and the National Heart, Lung, and Blood Institute (NHLBI) have set out to test adult modified transplantations, using thirty patients between the ages of 16–65 with SCD from 2004 through 2013.^52^ These clinical trials used modified bone marrow transplantation, a less toxic regimen to kill off some of their marrow cells.^52^ These patients underwent a stem cell transplant, receiving cells donated by a healthy brother or sister.^52^

Results were posted online in July 2014 by the *Journal of the American Medical Association*, where these clinical trials have discovered that the stem cell transplant reversed the disease in 26 of 30 patients (87%).^52^ These patients had normal hemoglobin, fewer hospital visits, and lower use of narcotics to treat pain from the disease^52,53^ and did not experience graft-versus-host disease.^52,53^ Fifteen patients successfully stopped immunosuppression medications a year after the transplant.^52,53^ The treatment was unsuccessful in only 4 patients with complications involving recurrent infections.^52,53^ Hematopoietic stem cell transplant (HSCT) has now become an important therapeutic option for patients with SCD; about 35 clinical trials at ClinicalTrials.gov are studying allogeneic bone marrow transplant (BMT) in patients with SCD. In an allogeneic transplant, the source of hematopoietic stem cells (HSCs) is from a donor (matched sibling, haploidentical family members, umbilical cord blood, or matched unrelated donor). BMT using HSCs from the latter 3 donor sources are risky; and donor availability is a limitation. These limitations can be overcome by an autologous transplant, in which the patient receives his cells after being modified by gene therapy. Genetically engineered autologous cells remove the need of finding a HSCT donor, hence making it available to all patients. Because they are the patient's stem cells, there is no need for immunosuppression, thereby eliminating the risks of graft versus host disease and immune-mediated graft rejection.^49^

#### 3.2.6 Transfusion therapy with complications

Chronic blood transfusions have been demonstrated to reduce the risk of both primary and secondary stroke and prevent repeated acute chest syndrome.^54^ Exchange transfusion therapy aims to reduce HbS to below 30%, which effectively prevents stroke.^55^ Chronic transfusions and exchange transfusions may lead to iron overload and iron deposition in organs, with end-organ damage.^55^ Therefore, it is important to reserve transfusion therapy for life-threatening complications.^55^ All patients requiring long-term transfusion therapy or those who have received multiple lifetime transfusions should be started on iron chelation therapy early and monitored closely for the detrimental effects of iron overload.^55,56^ Alloimmunization to red blood cell antigens is also a major complication of transfusion therapy and can be associated with delayed transfusion reactions characterized by fever, pain, dark urine, jaundice and pallor.^55^

An increase in the life expectancy of patients with sickle cell anemia in the US over 100 years was reported based on the findings that varying treatment regimens available, including hydroxyurea, prophylactic penicillin, and transfusion therapy, have proved to be effective in treating patients living with sickle cell anemia.^57^

## 4 FUTURE RESEARCH AND CONSIDERATIONS

### 4.1 SCD and public health

The World Health Organization estimates that over 40 countries are affected by Sickle cell disease but much of the data is hospital-based and not population-based.^58^ In regions where SCD is common, there are few adequately trained health professionals, available specialized health care facilities are insufficient, the medicine is often ineffective, and vaccines and safe blood transfusion are also very limited.^58^ Interventions for SCD such as stem cell transplantations, blood transfusions, and other gene modifying techniques are available in developed countries, however, these interventions are not benefiting a great majority of SCD patients, especially those with high mortality rates such as children and pregnant women.^58,59^ To improve the management of SCD, there is a crucial need for community education, genetic counseling, early case identification, and implementation of comprehensive health care management to those suffering from the disease.^58^

Benin, a country in West Africa, has successfully implemented a neonatal screening program for SCD.^58^ The program had a mortality rate 10 times lower for individuals under age 5 compared to the overall mortality rate of those with SCD under 5.^58^ These findings are consistent with those from developed countries, demonstrating the benefit of newborn screening and close follow-up of children using comprehensive health care management.^59^

### 4.2 Diagnostic methods: density-based separation

Standard diagnostic techniques in well-equipped laboratories, such as HE and HPLC, are too expensive and require more infrastructures than is available in many countries with high burdens of SCD, especially in rural areas.^60^ A newer technique to diagnose SCD may include density-based separation by aqueous multiphase systems (AMPSs).^60^ This specific technique identifies dense cells in the blood,^60^ which are cells that have *ρ* > 1.120 g/cm^3^ and are a characteristic of SCD.^60^ This technique also has a sensitivity of 90% (73%–98%) and a specificity of 97% (86%–100%).^60^ It has been created specifically for rural healthcare and the diagnosis timing including finger prick of the patient is less than 15 minutes, which is a great advantage over the other diagnostic tests which take anywhere from a few hours to several days to accurately receive patient results.^60^ Compared with currently available techniques, the density-based tests using AMPS are easy to use in rural areas and field medicine, they are of low cost, can diagnose rapidly, and have high sensitivity and specificity for SCD, potentially making them a better alternative.^60^

### 4.3 Stem cell research

Genetically engineered autologous cells provide more availability to patients by removing the need of finding a HSCs transplant donor. There is no need for immunosuppression since the patient's stem cells are used. Thereby eliminating the risks of graft versus host disease and immune-mediated graft rejection. Autologous hematopoietic stem cell transplant modification is done by gene editing or gene therapy.^49^ Dr Donald Kohn of the UCLA Eli and Edythe Broad Center for Regenerative Medicine and Stem Cell Research has conducted research and developed a technique that could offer patients suffering from SCD a one-time lasting treatment for SCD.^61^ Dr Kohn specially engineered enzymes, called zinc-finger nucleases (ZFNs), to eliminate the mutated genetic code and replace it with a corrected version that repairs the beta-globin mutation.^57^ By correcting the specific mutation in a patient's genetic code, this technique holds the potential to permanently treat sickle cell disease in the future.^61–63^

Many gene therapies based on gene addition using viral vectors to carry therapeutic genes in HSCs are in development with curative purposes. One such study that has reached clinical trials include a promising one where the patient's stem cells are infected with a lentivirus expressing an anti-sickling β-globin variant, T87Q. This vector is unique in that the amino acid substitution (β^A-T87Q^) allows for HPLC monitoring of the transgene globin levels in the patient's cells. Other gene therapies in the clinical trial are targeted towards fetal hemoglobin (HBF) induction as well as decreasing HBS production. Viral vectors, like lentivirus, are a great tool for gene therapy but their major limitations include: (a) their immunogenicity can create an inflammatory response in the donor thereby leading to a degeneration of the transducted tissue, (b) they can produce non-specific toxins, (c) based on the semi-random integration to the genome, a theoretical risk of insertional mutagenesis exist, (d) they possess limitations of transgenic capacity size. Hence the need for long-term monitoring of patients to evaluate both safety and efficacy.^49^

Gene-editing corrects a particular defective DNA in its location. Many studies are ongoing including ZFNs, transcription activator-like effector nucleases (TALENs), and the clustered regularly interspaced short palindromic repeat (CRISPR)-associated nuclease Cas9 approach which is the most advanced of the three. The CRISPRCas9 technology normally makes a double-stranded break in a specific genomic sequence directed to that site by a guide ribonuclease acid (RNA).^49^

### 4.4 Endothelin system: a therapeutic target for chronic pain

Children and adults with SCD experience recurrent and unpredictable episodes of pain due to vaso-occlusion.^64^ The endothelin pathway has been associated with vaso-occlusive pain as a trigger and a direct activator of nociceptors.^64^ Increased circulating ET-1 levels have been demonstrated clinically in sickle cell patients during vaso-occlusive episodes and these levels increase with increasing pain and decrease with decreasing pain.^47^ Besides, the endothelin system may also be involved in the sickling of red blood cells.^47^ ET-1 has been shown to play a role in modulating the activity of the Gardos channel, which is important in sickle erythrocyte dehydration via the ET_B_ receptor.^47^ Based on these studies, there is a need for a better understanding of the role of the endothelin system in SCD and the associated pain.^47,64^ This way, alternative treatments for SCD can be developed, providing more beneficial health care to the SCD community. Unraveling the pathophysiological targets of SCD has provided insights on clinical trials on anti-platelet and anti-adhesion agents, as well as anti-coagulation factors for the prevention of acute vaso-occlusive pain in SCD. One case in point is the development of an anti-P-selection molecule (Crizanlizumab) for treatment of sickle vaso-occlusive pain in adults and pediatric patients aged 16 and above, recently approved by the FDA in November 2019. Voxelotor (Oxbryta/GBT440) was also approved by the FDA in November 2019 for the treatment of SCD in adults and pediatric patients 12 years of age and above. Voxelotor binds to the N-terminus of the alpha subunit of HbS to stabilize the oxygenated hemoglobin state.^49^

### 4.5 Government-approved phytomedicines and nutraceuticals

Nicosan, formerly known as Niprisan, is an antisickling phytomedicine that has been reported to inhibit the polymerization of the hemoglobin S.^65^ It is a cocktail of 4 medicinal plants, *Piper guineense, Pterocapus osun, Eugenia caryophyllum,* and *Sorghum bicolor*, and is currently being marketed in Nigeria in encapsulated 250 mg/350 mg doses for a once-daily administration.^65^

Ciklavit primarily contains extracts of the plant *Cajanus Cajan*, essential amino acids, vitamins such as vitamin C, and minerals such as zinc.^65^ Ciklavit has also been reported to have anti-sickling properties, due to the presence of phenylalanine.^65^ The other role played by other components in Ciklavit is nutritional.^65^ Studies indicate that vitamin–mineral supplements of certain nutrients or treatment with a combination of high-dose antioxidants can reduce the percentage of irreversibly sickled cells.^65^ Zinc sulphate and magnesium help to reduce red blood cell dehydration which, in turn, prevents sickle cell crises, and reduces pain and complications.^65^ Furthermore, because low levels of zinc are found in SCD patients, with its known involvement in immune function, higher doses may help to decrease their susceptibility to infection.^66^ A study on children with sickle cell suggested that supplements may help improve growth and weight gain, boost the immune system, and protect against bacterial infections.^65^ In light of these studies and findings, it can be surmised that Ciklavit may cause a reduction in bone pains and ameliorate the adverse effects of sickle cell anemia on the liver.^65^ Therefore, Ciklavit may be a promising option for the treatment and management of sickle cell anemia.

Future research into alternative treatments have the beneficial potential to provide health care options to those who may not have access to them, nor can afford them.^67^ Therefore, the utilization of the most effective and appropriate therapies is of utmost importance in the management and maintenance of SCD.

## 5 CONCLUSION

With all the various symptoms and complications associated with SCD, early diagnosis and treatment are essential in preventing premature fatalities. By performing early diagnostic procedures such as newborn screening tests, as well as electrophoresis, and blood sample analysis, practical treatment methods can be arranged. Through prophylaxis therapy, pain management, administration of hydroxyurea, and other medications, along with transfusion therapy and performing transplants of the blood and bone marrow, patients have a chance to increase their longevity and maintain a semblance of a happy and healthy life.

## References

[1] Genetics Home Reference. Sickle cell disease [Internet]. Available at: http://ghr.nlm.nih.gov/condition/sickle-cell-disease. Accessed February 12, 2019.

[2] Maakaron J. Sickle Cell Anemia Clinical Presentation [Internet]. Medscape. Available at: http://emedicine.medscape.com/article/205926-clinical. Accessed February 12, 2019.

[3] Hopkinsmedicine.org. Sickle Cell Disease|Johns Hopkins Medicine Health Library [Internet]. Available at: http://www.hopkinsmedicine.org/healthlibrary/conditions/hematology_and_blood_disorders/sickle_cell_disease_85,P00101/. Accessed February 12, 2019.

[4] Gladwin MT Kato GJ Weiner D Nitric oxide for inhalation in the acute treatment of sickle cell pain crisis: a randomized controlled trial. *JAMA* 2011;305 (9):893–902. Available at: http://www.ncbi.nlm.nih.gov/pubmed/21364138. Accessed February 12, 2019 21364138 10.1001/jama.2011.235 PMC3403835

[5] Knott L. Sickle Cell Disease, Sickle Cell Anaemia [Internet]. Patient.co.uk. [updated 2017 Nov 1]. Available at: http://www.patient.co.uk/health/sickle-cell-disease-and-sickle-cell-anaemia-leaflet. Accessed February 12, 2019.

[6] Al-Salem AH. Splenic complications of sickle cell anemia and the role of splenectomy. *ISRN hematology*. 2010 Oct 31;2011. 10.5402/2011/864257. PMC3200071 22084706

[7] Bender MA. Sickle Cell Disease. 2003 Sep 15 [Updated 2017 Aug 17]. In: Adam MP, Ardinger HH, Pagon RA, et al., editors. GeneReviews® [Internet]. Seattle (WA): University of Washington, Seattle; 1993-2020. Available from: https://www.ncbi.nlm.nih.gov/books/NBK1377/. Accessed February 12, 2019.

[8] Cdc.gov. CDC—Sickle Cell Disease, Complications and Treatments—NCBDDD [Internet]. [Updated 2017 Aug 9]. Available at: http://www.cdc.gov/ncbddd/sicklecell/treatments.html. Accessed February 12, 2019.

[9] Rhodes M Akohoue SA Shankar SM Growth patterns in children with sickle cell anemia during puberty. *Pediatr Blood Cancer* 2009;53 (4):635–641. doi: 10.1002/pbc.22137. 19544390 10.1002/pbc.22137 PMC2733167

[10] Serjeant Graham R. The natural history of sickle cell disease. *Cold Spring Harb Perspect Med* 2013;a011783. doi: 10.1101/cshperspect.a011783. 23813607 10.1101/cshperspect.a011783 PMC3784812

[11] Grosse S Odame I Atrash H Amendah D Piel F Williams T. Sickle cell disease in Africa. *Am J Prev Med* 2011;41 (6):S398–S405. doi: 10.1016/j.amepre.2011.09.013. 22099364 10.1016/j.amepre.2011.09.013 PMC3708126

[12] Centers for Disease Control and Prevention: Sickle Cell Disease, Data, and Statistics [Internet]. 2011 [updated 2017 Aug 9]. Available at: http://www.cdc.gov/ncbddd/sicklecell/data.html. Accessed February 13, 2019.

[13] Aneni E Hamer D Gill C. Systematic review of current and emerging strategies for reducing morbidity from malaria in sickle cell disease. *Trop Med Int Health* 2013;18 (3):313–327. doi: 10.1111/tmi.12056. 23320577 10.1111/tmi.12056

[14] Williams TN Thein SL. Sickle cell anemia and its phenotypes. *Annu Rev Genomics Hum Genet* 2018;19:113–147. 29641911 10.1146/annurev-genom-083117-021320 PMC7613509

[15] Shriner D Rotimi CN. Whole-genome-sequence-based haplotypes reveal single origin of the sickle allele during the holocene wet phase. *Am J Hum Genet* 2018;102 (4):547–556. 29526279 10.1016/j.ajhg.2018.02.003 PMC5985360

[16] Jastaniah W. Epidemiology of sickle cell disease in Saudi Arabia. *Ann Saudi Med* 2011;31 (3):289. Available at: http://www.ncbi.nlm.nih.gov/pmc/articles/PMC3119971/. Accessed February 13, 2019 21623060 10.4103/0256-4947.81540 PMC3119971

[17] Orish VN Onyeabor OS Sanyaolu AO Iriemenam NC. Evaluating the knowledge of sickle cell disease and hemoglobin electrophoretic pattern among people living in Sekondi-Takoradi Metropolis, Ghana. *J Med Tropics* 2014;16 (2):56.

[18] Hassell KL. Population estimates of sickle cell disease in the US. *Am J Prev Med* 2010;38 (4):S512–S521. 20331952 10.1016/j.amepre.2009.12.022

[19] Bardakdjian-Michau J Bahuau M Hurtrel D Godart C Riou J Mathis M Neonatal screening for sickle cell disease in France. *J Clin Pathol* 2009;62 (1):31–33. doi: 10.1136/jcp.2008.058867. PMID 19103855. 19103855 10.1136/jcp.2008.058867

[20] Gyang E Yeom K Hoppe C Partap S Jeng M. Effect of chronic red cell transfusion therapy on vasculopathies and silent infarcts in patients with sickle cell disease. *Am J Hematol* 2010;86 (1):104–106. doi: 10.1002/ajh.21901. 10.1002/ajh.21901 21117059

[21] Nlm.nih.gov. Newborn Screening Tests: Medline Plus Medical Encyclopedia [Internet]. Available at: http://www.nlm.nih.gov/medlineplus/ency/article/007257.htm. Accessed February 13, 2019.

[22] Mayoclinic.org. Sickle Cell Anemia Tests and Diagnosis—Diseases and Conditions—Mayo Clinic [Internet]. [Updated 2018 March 8]. Available at: http://www.mayoclinic.org/diseases-conditions/sickle-cell-anemia/basics/tests-diagnosis/con-20019348. Accessed February 13, 2019.

[23] Labtestsonline.org. Sickle Cell Tests: The Test [Internet]. [Updated 2018 Dec 21]. Available at: http://labtestsonline.org/understanding/analytes/sickle/tab/test/. Accessed February 13, 2019.

[24] Lees C Davies SC Dezateux C. Neonatal screening for sickle cell disease. *Cochrane Database Syst Rev* 2000;(1). doi: 10.1002/14651858.CD001913. 10.1002/14651858.CD001913 PMC8406994 10796837

[25] Glassberg J. Evidence-based management of sickle cell disease in the emergency department. *Emerg Med Pract* 2011;13 (8):1–20. 22164362

[26] Nhlbi.nih.gov. How Is Hemolytic Anemia Diagnosed?—NHLBI, NIH [Internet]. Available at: http://www.nhlbi.nih.gov/health/health-topics/topics/ha/diagnosis. Accessed February 13, 2019.

[27] Anie KA Green J. Psychological therapies for sickle cell disease and pain. *Cochrane Database Syst Rev* 2015;(5):10.1002/14651858.CD001916.pub3 PMC7063720 25966336

[28] Kavanagh PL Sprinz PG Vinci SR Bauchner H Wang CJ. Management of children with sickle cell disease: a comprehensive review of the literature. *Pediatrics* 2011;128 (6):e1552–e1574. doi: 10.1542/peds.2010-3686. PMID 22123880. 22123880 10.1542/peds.2010-3686

[29] Ramirez I. Sickle Cell Anemia Skeletal Imaging [Internet]. Emedicine.medscape.com. 2013 [Updated 2017 March 30]. Available at: http://emedicine.medscape.com/article/413542-overview#a19. Accessed February 13, 2019.

[30] Krejza J Chen R Romanowicz G Sickle cell disease and transcranial Doppler imaging: inter-hemispheric differences in blood flow Doppler parameters. *Stroke* 2011;42 (1):81–86. 21088242 10.1161/STROKEAHA.110.591818 PMC3079337

[31] Sarkar S Ghosh S Ghosh S Collier A. Role of transcranial Doppler ultrasonography in stroke. *Postgrad Med J* 2007;83 (985):683–689. doi: 10.1136/pgmj.2007.058602. 17989267 10.1136/pgmj.2007.058602 PMC2659960

[32] Shatat IF Jakson SM Blue AE Johnson MA Orak JK Kalpatthi R. Masked hypertension is prevalent in children with sickle cell disease: a Midwest Pediatric Nephrology Consortium study. *Pediatr Nephrol* 2013;28 (1):115–120. 22886281 10.1007/s00467-012-2275-9

[33] Clarke GM Higgins TN. Laboratory investigation of hemoglobinopathies and thalassemias: review and update. *Clin Chem* 2000;46 (8):1284–1290. Available at: http://www.clinchem.org/content/46/8/1284.long. Accessed February 13, 2019 10926923

[34] Angastiniotis M, Eleftheriou A, Galanello R, Harteveld CL, Petrou M, Traeger-Synodinos J, Giordano P, Jauniaux E, Modell B, Serour G. Conventional prenatal diagnosis. In: Old J, ed. *Prevention of Thalassaemias and Other Haemoglobin Disorders*. 2nd ed. Available at: http://www.ncbi.nlm.nih.gov/books/NBK190475/. Accessed February 13, 2019.

[35] NIH, National Heart, Lung, and Blood Institute. June 2002, 4th ed.; Management and Treatment of Sickle Cell Disease. https://www.nhlbi.nih.gov/files/docs/guidelines/sc_mngt.pdf. Accessed February 13, 2019.

[36] Serjeant GR. Sickle-cell disease. *Lancet* 1997;350 (9079):725–730. Available at: http://scinfo.org/wp-content/uploads/2016/02/sickle-cell-disease.pdf. Accessed February 13, 2019 9291916 10.1016/S0140-6736(97)07330-3

[37] Adams-Graves P Bronte-Jordan L. Recent treatment guidelines for managing adult patients with sickle cell disease: challenges in access to care, social issues, and adherence. *Expert Rev Hematol* 2016;9 (6):541–552. 27098013 10.1080/17474086.2016.1180242

[38] Evidence-Based Management of Sickle Cell Disease. Expert Panel Report, 2014. National Institutes of Health. National Heart, Lung, and Blood Institute, 2014. Available at: https://www.nhlbi.nih.gov/health-topics/evidence-based-management-sickle-cell-disease. Accessed February 13, 2019.

[39] Brousse V Buffet P Rees D. The spleen and sickle cell disease: the sick (led) spleen. *Br J Haematol* 2014;166 (2):165–176. doi: 10.1111/bjh.12950. 24862308 10.1111/bjh.12950

[40] Ndefo U Maxwell A Nguyen H Chiobi T. Pharmacological management of sickle cell disease. *P T* 2008;33 (4):238–243. 19750169 PMC2730092

[41] Gatson MH Verter JI Woods G Prophylaxis with oral penicillin in children with sickle cell anemia: A randomized trial. *N Engl J Med* 1986;314:1593–1599. 3086721 10.1056/NEJM198606193142501

[42] Falletta JM Woods GM Verter JI Discontinuing penicillin prophylaxis in children with sickle cell anemia. *J Pediatrics* 1995;127 (5):685–690. Available at: http://www.ncbi.nlm.nih.gov/pubmed/7472817/. Accessed February 13, 2019 10.1016/s0022-3476(95)70154-0 7472817

[43] Cober MP Phelps SJ. Penicillin prophylaxis in children with sickle cell disease. *J Pediatr Pharmacol Ther* 2010;15 (3):152–159. Available at: http://www.ncbi.nlm.nih.gov/pmc/articles/PMC3018247/. Accessed on February 13, 2019 22477807 PMC3018247

[44] Pai VB Nahata MC. Duration of penicillin prophylaxis in sickle cell anemia: issues and controversies. *Pharmacotherapy* 2000;20 (1):110–117. 10641985 10.1592/phco.20.1.110.34660

[45] Brun M Bourdoulous S Couraud PO Elion J Krishnamoorthy R Lapoumeroulie C. Hydroxyurea downregulates endothelin-1 gene expression and upregulates ICAM-1 gene expression in cultured human endothelial cells. *Pharmacogenomics J* 2003;3 (4):215. 12931135 10.1038/sj.tpj.6500176

[46] Johnson C, Telen MJ. Adhesion molecules and hydroxyurea in the pathophysiology of sickle cell disease. 2008:481–485. 10.3324/haematol.12734 18379005

[47] Smith T Haymond T Smith S Sweitzer S. Evidence for the endothelin system as an emerging therapeutic target for the treatment of chronic pain. *J Pain Res* 2014;7:531–545. 25210474 10.2147/JPR.S65923 PMC4155994

[48] Claster S Vichinsky EP. Managing sickle cell disease. *BMJ* 2003;327 (7424):1151–1155. 14615343 10.1136/bmj.327.7424.1151 PMC261819

[49] Salinas Cisneros G Thein SL. Recent advances in the treatment of sickle cell disease. *Front Physiol* 2020;11:435. 32508672 10.3389/fphys.2020.00435 PMC7252227

[50] Walters MC Storb R Patience M Impact of bone marrow transplantation for symptomatic sickle cell disease: an interim report. *Blood* 2000;95 (6):1918–1924. Available at: http://www.bloodjournal.org/content/95/6/1918?sso-checked=true. 10706855

[51] Ratko TA, Belinson SE, Brown HM, Noorani HZ, Chopra RD, Marbella A, Samson DJ, Bonnell CJ, Ziegler KM, Aronson N. Hematopoietic stem-cell transplantation in the pediatric population. 2012. Available at: http://www.ncbi.nlm.nih.gov/books/NBK84626/. Accessed on February 13, 2019.

[52] National Institutes of Health. Stem Cell Transplant Reverses Sickle Cell Disease in Adults [Internet]. Available at: http://www.nih.gov/researchmatters/july2014/07142014sickle.htm. Accessed February 13, 2019.

[53] Hsieh M Fitzhugh C Weitzel R Link M Coles W Zhao X Nonmyeloablative HLA-matched sibling allogeneic hematopoietic stem cell transplantation for severe sickle cell phenotype. *JAMA* 2014;312 (1):48. 25058217 10.1001/jama.2014.7192 PMC4698790

[54] Chou ST. Transfusion therapy for sickle cell disease: a balancing act. *ASH Edu Program* 2013;2013 (1):439–446. Available at: http://asheducationbook.hematologylibrary.org/content/2013/1/439.full.pdf. 10.1182/asheducation-2013.1.439 24319217

[55] Kanter J Kruse-Jarres R. Management of sickle cell disease from childhood through adulthood. *Blood Rev* 2013;27 (6):279–287. DOI: 10.1016/j.blre.2013.09.001. Accessed on February 13, 2019 24094945

[56] Roseff SD. Sickle cell disease: a review. *Immunohematology* 2009;25 (2):67–74. Available at: http://europepmc.org/abstract/med/19927623. 19927623

[57] Nhlbi.nih.gov. Reducing the Burden of Sickle Cell Disease—NHLBI, NIH [Internet]. Available at: http://www.nhlbi.nih.gov/news/spotlight/success/reducing-burden-sickle-cell-disease. Accessed February 13, 2019.

[58] World Health Organization. (2010). *Sickle-Cell Disease: A Strategy for the WHO African Region* (AFR/RC60/8). Washington, DC: Government Printing Office.

[59] Villers MS Jamison MG De Castro LM James AH. Morbidity associated with sickle cell disease in pregnancy. *Am J Obstet Gynecol* 2008;199:125.e1–125.e5. 18533123 10.1016/j.ajog.2008.04.016

[60] Kumar AA Patton MR Hennek JW Density-based separation in multiphase systems provides a simple method to identify sickle cell disease. *Proc Natl Acad Sci U S A* 2014;111 (41):14864–14869. doi: 10.1073/pnas.1414739111. 25197072 10.1073/pnas.1414739111 PMC4205650

[61] Vogt-James M. Stem Cell Researchers Develop Promising Method to Treat Sickle Cell Disease [Internet]. Medicalxpress.com. 2015. Available at: http://medicalxpress.com/news/2015-03-stem-cell-method-sickle-disease.html. Accessed February 13, 2019.

[62] Gammon K. Gene therapy: editorial control. *Nature* 2014;515 (7526):S11–S13. doi: 10.1038/515s11a. 25390136 10.1038/515S11a

[63] Romero Z Urbinati F Geiger S β-Globin gene transfer to human bone marrow for sickle cell disease. *J Clin Investig* 2013;123 (8):3317–3330. 10.1172/JCI67930 PMC4011030 23863630

[64] Smith WR Scherer M. Sickle-cell pain: advances in epidemiology and etiology. *ASH Educ Program* 2010;2010 (1):409–415. doi: 10.1182/asheducation-2010.1.409. 10.1182/asheducation-2010.1.409 21239827

[65] Imaga NA. Phytomedicines, and nutraceuticals: alternative therapeutics for sickle cell anemia. *Sci World J* 2013;2013 (269659). doi:10.1155/2013/269659. 10.1155/2013/269659 PMC3586489 23476125

[66] Booth C Inusa B Obaro SK. Infection in sickle cell disease: a review. *Int J Infect Dis* 2010;14 (1):e2–e12. doi: 10.1016/j.ijid.2009.03.010. 10.1016/j.ijid.2009.03.010 19497774

[67] Hoots WK Shurin SB. Future directions of sickle cell disease research: the NIH perspective. *Pediatr Blood Cancer* 2012;59 (2):353–357. doi: 10.1002/pbc.24180. Available at: http://www.ncbi.nlm.nih.gov/pmc/articles/PMC3374062/. Accessed February 13, 2019 22517801 10.1002/pbc.24180 PMC3374062

